# Intracortical Bone Remodeling Variation Shows Strong Genetic Effects

**DOI:** 10.1007/s00223-013-9775-x

**Published:** 2013-08-27

**Authors:** L. M. Havill, M. R. Allen, J. A. K. Harris, S. M. Levine, H. B. Coan, M. C. Mahaney, D. P. Nicolella

**Affiliations:** 1 Department of Genetics, Texas Biomedical Research Institute, San Antonio, TX 78227 USA; 2 Department of Anatomy and Cell Biology, Indiana University School of Medicine, Indianapolis, IN 46202 USA; 3 Southwest National Primate Research Center, San Antonio, TX 78227 USA; 4 Mechanical and Materials Engineering, Southwest Research Institute®, San Antonio, TX 78228 USA

**Keywords:** Primate, Osteoporosis, Biomechanics, Population studies, Bone histomorphometry

## Abstract

Intracortical microstructure influences crack propagation and arrest within bone cortex. Genetic variation in intracortical remodeling may contribute to mechanical integrity and, therefore, fracture risk. Our aim was to determine the degree to which normal population-level variation in intracortical microstructure is due to genetic variation. We examined right femurs from 101 baboons (74 females, 27 males; aged 7–33 years) from a single, extended pedigree to determine osteon number, osteon area (On.Ar), haversian canal area, osteon population density, percent osteonal bone (%On.B), wall thickness (W.Th), and cortical porosity (Ct.Po). Through evaluation of the covariance in intracortical properties between pairs of relatives, we quantified the contribution of additive genetic effects (heritability [*h* ^2^]) to variation in these traits using a variance decomposition approach. Significant age and sex effects account for 9 % (Ct.Po) to 21 % (W.Th) of intracortical microstructural variation. After accounting for age and sex, significant genetic effects are evident for On.Ar (*h* ^2^ = 0.79, *p* = 0.002), %On.B (*h* ^2^ = 0.82, *p* = 0.003), and W.Th (*h* ^2^ = 0.61, *p* = 0.013), indicating that 61–82 % of the residual variation (after accounting for age and sex effects) is due to additive genetic effects. This corresponds to 48–75 % of the total phenotypic variance. Our results demonstrate that normal, population-level variation in cortical microstructure is significantly influenced by genes. As a critical mediator of crack behavior in bone cortex, intracortical microstructural variation provides another mechanism through which genetic variation may affect fracture risk.

## Introduction

Histomorphological structures that result from intracortical bone remodeling are critical determinants of bone’s resistance to fracture. These structures and the resulting variation in the degree and heterogeneity of mineralization influence crack propagation and/or energy dissipation through the bone cortex. While bone mass and trabecular bone architecture undoubtedly play essential and synergistic roles in bone fragility, recent research has brought the role of cortical bone and intracortical microstructure in bone fracture resistance to the forefront [1].

Comparisons of individuals with femoral neck fracture to age-matched controls reveal strong associations between intracortical remodeling variation and fracture incidence. For example, porosity is consistently greater in the fracture groups [2–4], as are haversian (central) canal size and density [4–6]. Research also shows smaller numbers of osteons per unit area (but with larger canals) [4, 6], larger osteons [6], higher rates of osteonal remodeling [2], and spatial clustering of osteons [7] producing “giant” (in the top 0.05 % of size in controls) canals [3] in fracture groups. These data are all consistent with the idea that bone turnover rate, and by association the structures resulting from turnover, can significantly affect fracture risk independently of bone mineral density (BMD) [8, 9].

Intracortical (osteonal) remodeling contributes substantially to determining bone strength and energy to failure, but the relationship between the presence of osteons and bone mechanical properties is not simple. Histomorphological outcomes of osteonal remodeling account for 49–68 % of fracture toughness variation in the human femur [10]. Intracortical remodeling results in increased bone porosity due to a higher number of central canals, which can weaken bone. The osteons themselves, though, can act to deflect cracks and increase bone’s fracture toughness [10–12]. Newer, more ductile osteons act to toughen bone (making it more resistant to fracture); but older, more brittle osteons may have the opposite effect [12, 13]. Additionally, the effect of osteons on bone resistance to fracture depends upon the type of stress being applied and the orientation of collagen fibers within the osteons [14, 15]. Many details of the relationship between intracortical remodeling and bone fracture resistance are still being elucidated, but it is unequivocal that these features of cortical bone microstructure affect bone fragility and resistance to crack initiation and growth [16].

Based on research with other bone traits, we [17] and others [18] have hypothesized that genetic variation may be responsible for much of the observed variation in intracortical histomorphology, thereby providing an avenue for the investigation and identification of bone fragility genes. Studies examining correlations between bone phenotypes among related individuals provide unequivocal evidence that heritability is an important component of bone metabolism variation, but none of the hundreds of studies of genetic effects on skeletal mass and turnover have specifically addressed the heritability of intracortical remodeling variation. This is primarily because structures necessary to assess intracortical morphology cannot be visualized with in vivo imaging of humans, coupled with the fact that rodent models—often used for genetic studies—lack intracortical remodeling under normal conditions. Data exist from only one study, in a nonhuman primate model, that suggest that at least two intracortical remodeling variables, osteon size, and haversian canal size are influenced by genetic variation [17].

The specific aim of this study was to test the hypothesis that there is a significant genetic effect on variation in histomorphological outcomes of intracortical remodeling using an outbred nonhuman primate model. Additionally, we aimed to estimate the magnitude of any detectable genetic effect in this population and to identify the histomorphometric traits in which genetic effects are most strongly reflected. We conducted this study in the baboon, a well-established nonhuman primate model in skeletal genetics [19–25].

## Methods

**Fig. 1:**
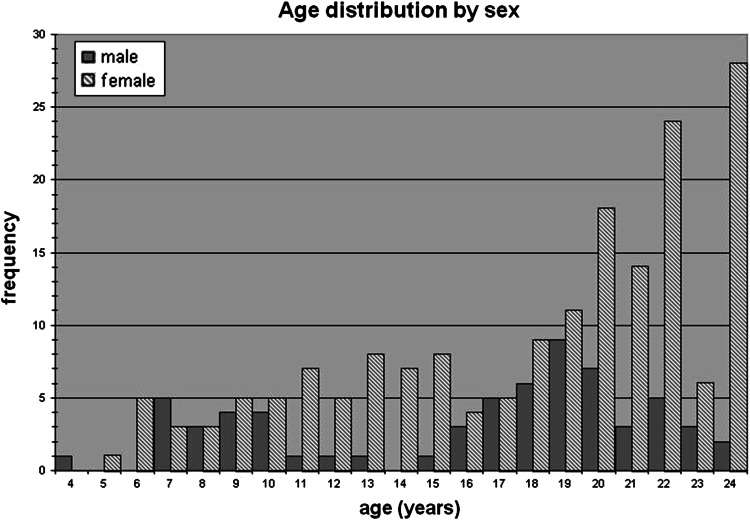
Age distribution of the sample constituents by sex

Right femurs from 101 baboons, all members of a single, extended pedigree, were obtained at necropsy from 74 females and 27 males ranging in age from 7 to 33 years (approximately developmentally equivalent to 21–99 years in humans). Figure 1 shows the age distribution of the sample by sex. During life all animals were housed outdoors in social group housing and maintained on commercial monkey chow to which they had ad libitum access. Animal care personnel and staff veterinarians provided daily maintenance and health care to all animals. All procedures related to their treatment during their lives at the Texas Biomedical Research Institute (TBRI)/Southwest National Primate Research Center (SNPRC) were approved by the Institutional Animal Care and Use Committee in accordance with established guidelines. All animals were sacrificed for reasons unrelated to this project or died naturally. Complete clinical records for each animal were checked to be certain that animals with medical conditions known to affect bone metabolism (e.g., rheumatoid arthritis, diabetes, chronic renal disease) were not included in the sample. Reliable fracture history and/or fracture risk data are not available for these baboons.

### Baboon Pedigree

The baboons were members of a single six-generation pedigree that includes a total of 2,426 individuals. This pedigree, with 384 founders, has resulted from managed breeding of baboons at the SNPRC. In this complex pedigree, full sibships range in size from 2 (*n* = 372) to 12 (*n* = 10), with a median of 5. Fifty additional classes of relative pairs are represented in this extended pedigree. Examples of these pair classes included parent-offspring (*n* = 350), half-sibling (*n* = 6,855), half-avuncular (*n* = 6,414), and double half-first cousin (*n* = 754) pairs.

A subset of 101 baboons was used in the present study. This subset exhibits a rich array of relative pairs in what essentially is a single extended family. This includes those that are related at the degree of parent–offspring (*n* = 3), full siblings (*n* = 10), avunculars (e.g., uncle/aunt:nephew/niece; *n* = 8), half-siblings (*n* = 153), first cousins (*n* = 73), and first cousins once removed (*n* = 22).

### Data Collection

**Fig. 2:**
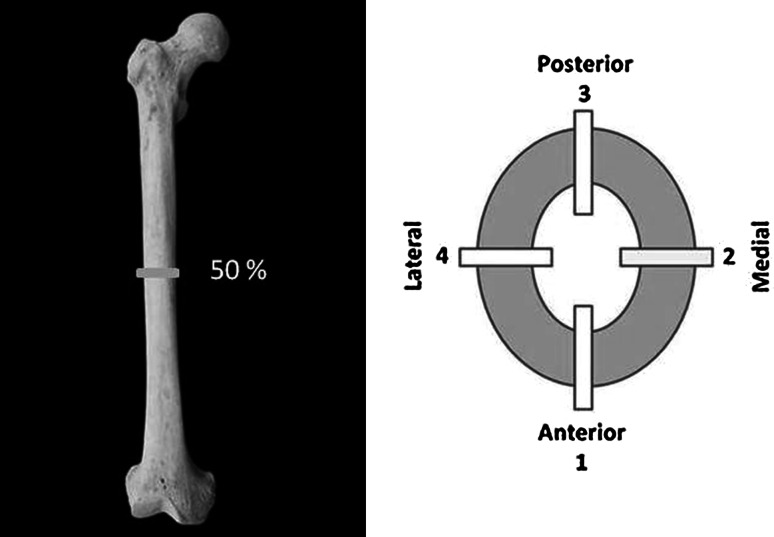
Sampling location on baboon femur and indication of rays along which data were collected

Femurs were collected at necropsy, wrapped in saline-soaked gauze, placed in air-tight plastic bags, and frozen until preparation for testing. One section, ~300 μm thick, was cut from the midshaft femur using an Isomet 1000 Precision Saw (Buehler, Lake Bluff, IL), then ground manually to ~100 μm. Sections were stained with toluidine blue and analyzed under bright field illumination using an Olympus (Center Valley, PA) BX41 laboratory microscope with attached Q-imaging Qicam Fast 1394 camera. Direct measures of intracortical remodeling dynamics were obtained using Bioquant Osteo v. 7.20 (R&M Biometrics, Nashville, TN). All measurements were recorded via manual selection and/or tracing. The cortex was sampled by reading along four rays (1, anterior; 2, lateral; 3, posterior; 4, medial) running from the endosteal to the periosteal surface at 100× magnification (Fig. 2).

Standard methods for characterizing cortical bone microstructure in the absence of in vivo fluorochrome labeling were used to measure the following histomorphometric variables related to intracortical remodeling [26, 27]. Whenever possible, measurement names and abbreviations of such adhere to the system of nomenclature, standards, and units described in Parfitt et al. [28].

1. Osteon area (μm^2^, On.Ar): The total area circumscribed by the reversal (cement) line averaged across all osteons within the field of assessment
2. Haversian (central) canal area (μm^2^, H.Ar): The total area of the haversian canal averaged across all canals within the field of assessment
3. Osteon population density (#/mm^2^, OPD), (osteon number + osteon fragment number)/bone area: The number of secondary osteons with intact haversian canals plus the number of osteon fragments (the number of secondary osteons in the field of view that are without intact haversian canals) normalized by the total bone tissue assessed
4. Percent osteonal bone (%On.B), (osteonal area/bone area) × 100: The proportion of the observed cortex occupied by secondary osteons with intact haversian canals
5. Wall thickness (μm, W.Th): The average thickness of the wall of the osteon (between the reversal line and the central canal perimeter)
6. Cortical porosity (%, Ct.Po), (void area/bone area) × 100: The ratio of intracortical void area (including central and longitudinal canals but excluding osteocyte lacunae) to total bone area.

Ten percent of specimens were remeasured to determine coefficients of variation for the direct measurements: On.Ar, 2.9 %; W.Th, 3.1 %, H.Ar, 2.2 %, pore area (excluding central canals), 7.1 %; bone area, 3.3 %.

### Statistical and Quantitative Genetic Analysis

Age, sex, and additive genetic effects on variation in intracortical histomorphometry were assessed using maximum likelihood–based variance components methods. Evaluation of the covariance in intracortical properties between pairs of relatives allows for quantification of the contribution of additive genetic effects (heritability [*h* ^2^]) to variation in these traits. In this case relative pairs are pairs of baboons for which measures of biological relationship are a function of the probabilities of genetic similarity—because of inheritance of allelic forms of genes or variants within them from a common ancestor—at a locus in the two genomes of a pair of individuals.

We used a variance decomposition approach, implemented in the computer package SOLAR (described in detail elsewhere [29]), to simultaneously estimate *h* ^2^—the proportion of the total phenotypic variance (variance in the trait of interest) that is attributable to additive genetic effects. This approach involves modeling the expected phenotypic covariance among relatives as $\hat{\Upomega } = 2\Upphi \sigma_{\text{G}}^{2} + I\sigma_{\text{E}}^{2}$, where $2\Upphi \sigma_{\text{G}}^{2}$, the additive genetic component, is the product of two times the kinship matrix ($\Upphi$) and the additive genetic variance ($\sigma_{\text{G}}^{2}$) and $I\sigma_{\text{E}}^{2}$, the unique environmental component, is the product of the identity matrix (*I*) and the non-genetic variance component ($\sigma_{\text{E}}^{2}$). The phenotypic variance ($\sigma_{\text{P}}^{2}$) is thereby partitioned into its additive genetic ($\sigma_{\text{G}}^{2}$) and environmental ($\sigma_{\text{E}}^{2}$) components, allowing for estimation of the proportion of the phenotypic variance attributable to additive genetic effects (i.e., *h* ^2^) as $h^{2} = {{\sigma_{\text{G}}^{2} } \mathord{\left/ {\vphantom {{\sigma_{\text{G}}^{2} } {\sigma_{\text{P}}^{2} }}} \right. \kern-0pt} {\sigma_{\text{P}}^{2} }}$ and that proportion attributable to non-genetic factors as $e^{2} = 1 - h^{2}$.

Age and sex terms were selected for inclusion as covariates in the final models by means of a Bayesian model averaging procedure implemented in SOLAR. This procedure allows for the evaluation of all possible covariates (age, age^2^, sex, sex-by-age, and sex-by-age^2^) alone and in all possible combinations to identify the best set for inclusion based on a Bayesian information criterion for each covariate/combination and a posterior probability assigned to each covariate [30].

Ultimately, phenotypes were modeled as $y = \mu + \beta_{1} x_{1} + \beta_{2} x_{2} + \cdots + \beta_{n} x_{n} + g + e$, where *μ* is the population mean for the trait, *x*_*i*_ are the values of significant age and sex covariates, *β*_*i*_ are their mean effects coefficients, and *g* and *e*, respectively, are the genetic and environmental effects.

The significance of maximum likelihood estimates for *h* ^2^ and other parameters was assessed by means of likelihood ratio tests. The maximum likelihood for the general model in which all parameters are estimated was compared to that for a restricted model in which the value of the parameter to be tested is held constant at some value (usually zero). Twice the difference in the ln likelihoods of the two models is distributed asymptotically approximately as either a 1/2:1/2 mixture of *χ* ^2^ and a point mass at zero for tests of parameters like *h* ^2^ (for which a value of zero in a restricted model is at a parameter space boundary) or an *χ* ^2^ variate for tests of covariates (for which zero is not a boundary value). Degrees of freedom equal the difference in the number of estimated parameters in the two models. However, for tests of parameters like *h* ^2^, whose values may be fixed at a boundary of their parameter space in the null model, the appropriate significance level is obtained by halving the *p* value.

## Results

### General Population Variation

Table 1 shows descriptive statistics by sex for each of the quantitative variables. Although not a primary focus of this study, *t* tests and resulting *p* values for differences between the sexes are presented to give a general appreciation for sex variability in this sample. A Bonferroni correction (modified to account for correlation among the traits) results in a required *p* value of 0.015 for significance. Nonetheless, because these data derive from related individuals, statistical tests that do not account for this fact (e.g., Student’s *t* tests) must be interpreted with caution. The results of the maximum likelihood–based tests (that do account for relatedness among individuals) for significant effects of all age and sex terms are presented in Table 2.

**Table 1:** Descriptive statistics for intracortical remodeling dynamics by sex

Variable	Males (*n* = 27)			Females (*n* = 74)			*p*
	Range	Mean	SD	Range	Mean	SD	
Age (years)	7.03–25.51	17.39	5.06	7.33–33.27	20.18	5.72	0.028
On.Ar (μm^2^)	14,602.86–37,154.29	22,472.28	6,070.74	10,654.64–27,040.97	19,909.35	3,667.27	0.047
H.Ar (μm^2^)	916.66–2,510.30	1,531.74	400.00	716.42–4,054.94	1,906.37	677.37	0.001*
OPD (#/mm^2^)	2.05–8.70	5.25	1.78	0.83–17.00	6.14	2.90	0.140
%On.B	4.53–17.03	11.16	3.53	1.18–27.90	12.06	5.40	0.341
W.Th (μm)	45.78–78.54	62.86	8.32	39.23–75.20	57.82	8.23	0.008*
Porosity (%)	1.68–12.18	4.53	2.67	1.63–13.50	4.87	2.21	0.519

* Statistically significant at or below adjusted *p* value of 0.015

**Table 2:** Results of quantitative genetic analysis of osteon remodeling dynamics (with variables for which *h* ^2^ is statistically significant indicated in bold)

	Covariates	Total variation due to covariates	Residual variation	*h* ^2^ ± SE (*p*)	Total variation due to genes
**On.Ar (μm** ^**2**^ **)**	**Age-by-sex**	**0.20**	**0.80**	**0.79** **±** 0.33 (0.002)*	**0.63**
H.Ar (μm^2^)	Sex, age^2^	0.13	0.87	0.06 ± 0.26 (0.406)	0.05
OPD (#/mm^2^)	Age	0.18	0.82	0.29 ± 0.32 (0.139)	0.24
**%** **On.B**	**Age**	**0.09**	**0.91**	**0.82** **±** 0.33 (0.003)*	**0.75**
**W.Th** (μm)	**Age, age** ^**2**^	**0.21**	**0.79**	**0.61** **±** 0.36 (0.013)*	**0.48**
Porosity	Age^2^	0.09	0.91	0.25 ± 0.31 (0.172)	0.23

* Statistically significant at or below adjusted *p* value of 0.015

The mean age of females is older than that of males, but this difference does not reach statistical significance when the Bonferroni correction is applied. It is important to note that females are much better represented than males in the oldest age groups and are the only sex represented above the age of 26 years (Fig. 1). Significant sex differences include larger haversian canals in females (*p* = 0.001) and greater W.Th in males (*p* = 0.008). Mean osteon size trends toward larger in males but does not reach statistical significance after correction for multiple testing. The percentage of remodeled cortex (%On.B), OPD, and porosity showed no sex effect.

**Fig. 3:**
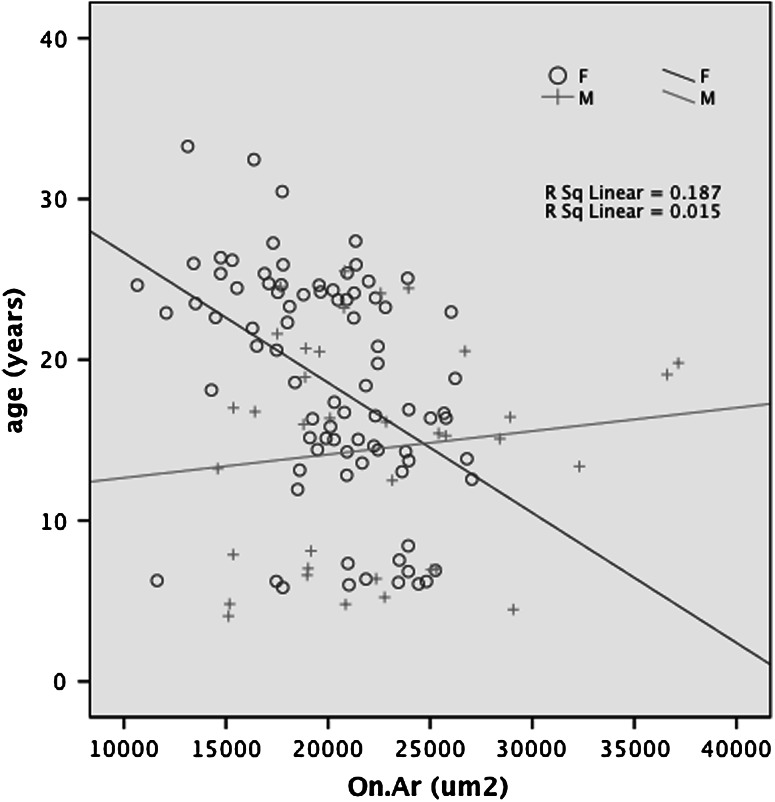
Scatterplot of osteon area (On.Ar) by age for both sexes with trend lines for males and for females

In Table 2 the results of the maximum likelihood–based Bayesian model averaging procedure to identify the best set of covariates for explaining variance in the trait of interest are presented. The sex trend of larger osteon size (On.Ar) in males is confirmed and is age-specific (sex-by-age), involving a decrease in osteon size with age in females that is not present in males (Fig. 3). This sex-specific age effect accounts for 20 % of the total phenotypic variance in osteon size. H.Ar demonstrates a significant sex effect, with a larger overall value in females, as well as an age-by-sex effect, with increasing H.Ar with increasing age in females but not in males. Together these effects explain 13 % of the variation in canal size. Significant and positive mean effects of age occur in OPD, %On.B, and porosity, accounting for 9–18 % of the total variance in these traits. A slight negative mean effect of age terms, driven largely by smaller values in older animals that are mostly females, explains 21 % of the variance in W.Th.

Investigations of patterns of variability in femoral intracortical microstructure in humans have produced varied results. Good, concise reviews of this somewhat contradictory literature are available elsewhere [31, 32]. Studies of osteon and haversian canal size predominate and attribute variability to ancestry/population affinity, age, physical activity and biomechanical factors, and sex; but clear and reliable patterns are difficult to discern. Some of the inconsistency in results is likely attributable to the fact that the effects of age and sex on intracortical microstructural variables can differ by cortical region (e.g., between the periosteal and endosteal aspects) [33] and that methods for sampling the cortex have not been standardized. In a sample size very similar to ours, Britz et al. [32] examined osteon size and shape in 88 human femurs obtained from cadavers. Their data collection was limited to the anterior third of the femoral cortex but revealed decreasing osteon size with increasing age as the dominant pattern of variation, consistent with the pattern we observed in the baboon mean On.Ar. As is clear from their result and their comprehensive review of the literature, sex does not appear to exert a strong, consistent effect on osteon size, which is also consistent with our observations in the baboon.

### Effects of Genes

Residual heritability estimates are presented in Table 2. As above, a modified Bonferroni correction that accounts for correlation among the traits results in a required *p* value of 0.015 for significance. These estimates show strong and significant genetic effects for On.Ar, %On.B, and mean W.Th. Seventy-nine percent (*p* = 0.002) of the 80 % of the total phenotypic variance that remains after accounting for the significant age-by-sex effect on On.Ar variance is attributable to the effects of genes. This translates to a genetic effect accounting for 63 % of the total phenotypic variation in On.Ar in these baboons. Table 2 presents these figures and similar results for %On.B and mean W.Th. The residual heritability estimates of 0.82 (*p* = 0.003) for %On.B and 0.61 (*p* = 0.013) for W.Th translate to genetic effects that account for 75 and 48 % of the *total* phenotypic variance in amount of remodeled cortex and mean wall thickness, respectively.

## Discussion

Our results support the hypothesis that genetic background contributes substantially to population-level variation in the intracortical remodeling process and resulting intracortical microstructure. On.Ar, %On.B, and mean W.Th show strong genetic effects (residual *h* ^2^ ranging 0.61–0.82) in this nonhuman primate model for human bone maintenance and turnover. These heritability estimates translate to genetic effects that account for 48–75 % of the total trait variance in these baboons. For comparison, age and sex effects account for 9–21 % of the trait variance. Because we know intracortical remodeling plays a role in bone fragility, the magnitude of the genetic effects on On.Ar, %On.B, and W.Th suggest that the specific genes that drive variation in intracortical remodeling are potentially important contributors to bone fragility. Our results also indicate that outbred primate populations such as these baboons can serve as valuable study populations in which to identify these genes. Furthermore, our results indicate that On.Ar, W.Th, and %On.B may be the most fruitful parameters on which to focus as they appear to yield the strongest genetic signal.

It is important to note that heritability estimates are population-specific and that we would not expect the magnitude of the additive effect of genes in this baboon population to translate directly to any population of humans. We would expect, however, as we have seen with a wide array of other bone-related phenotypes, that those traits that are heritable in one species are heritable in the other. Furthermore, traits yielding moderate but nonsignificant heritability estimates (i.e., OPD, porosity) should not be dismissed for future study on the basis of our results. Failure to achieve statistical significance may be due to large standard errors around our estimates that would resolve with a larger sample size.

Cortical bone strength and fracture toughness are strongly related to cortical microstructure, particularly osteon size and density, and intracortical porosity [10, 34–36]. Fracture toughness, which is a measure of the ability of bone tissue to resist initiation and propagation to failure of a microcrack, is highly dependent upon cortical bone microstructure. One of the primary fracture toughening mechanisms in cortical bone is crack deflection by osteons [12, 16, 37–39]. Propagating microcracks are deflected by osteons more often in bone from young individuals than in bone from old individuals. The result is significantly greater fracture resistance in young individuals’ bone [12]. Crack deflection and resulting fracture resistance are controlled primarily by the local morphology and material properties of interstitial bone, osteons, cement lines, and local bone tissue porosity (predominantly haversian canal size and number) [13]. Fracture resistance in cortical bone significantly decreases with age, largely due to the inability of osteons to deflect microcracks [12]. In light of our study results, the ability of cortical bone to resist fracture and the decrease in fracture resistance with age may be strongly influenced by genetic factures that influence intracortical remodeling and, consequently, intracortical histomorphology.

It is well appreciated that women are at significantly increased fracture risk during the postmenopausal years, which is believed to be due primarily to the increased remodeling rate that ensues following declines in endogenous estrogen. These increased rates of remodeling are independently associated with fracture risk after controlling for BMD [8, 9]. Females are well represented throughout the life span in our sample. Our results show that females overall, relative to males, have larger haversian canals and smaller osteons. With increasing age, females show still smaller osteons and still larger haversian canals. Additionally, they show increases in OPD, %On.B, and porosity in conjunction with decreasing W.Th. A demonstrated genetic component to some of these microstructural parameters may help explain why certain females are particularly at risk for fracture.

This work is especially important in light of apparently rare but serious adverse conditions associated with potent remodeling suppression with some antiresorptive drugs (e.g., bisphosphonates and denosumab). We and others have hypothesized that genetic variation may underlie heterogeneity in intracortical remodeling rates and outcomes, which may, in turn, provide a potential mechanism to explain variation in the efficacy of antiresorptive drugs as therapy for bone fragility. Central to this line of inquiry is a test of the hypothesis that genetic variation may be responsible for intracortical remodeling variation, a hypothesis that our results strongly support. Further, our results indicate that osteon size, percent remodeled cortex, and osteon wall width are the histomorphological outcomes most strongly affected by variation in the bone metabolic processes that are influenced by the detected genetic effect. This supports the potential for a scenario in which certain individuals who are genetically predisposed to cortical microstructure that is less mechanically advantageous may experience disadvantageous responses to remodeling suppression such as being at higher risk for atypical femoral fractures. Further study will be necessary to specifically address this concept.

Although the study design for this initial test of the genetic hypothesis does not allow for identification of particular genes or genomic regions that are responsible for the effect, some information about the way these genes might act to produce variation in intracortical bone microstructure may be gleaned by examining the three traits in which genetic effects were detected and, to some extent, their relationship to the traits that showed no genetic effect. Osteon size is reflective of osteoclast action in that activity of these cells dictates the size of the space that is remodeled by a bone multicellular unit (BMU); however, osteocytes may indirectly affect osteon size by signaling the osteoclasts to stop resorbing bone [40, 41]. In this way, genes that affect osteocyte sensitivity or signaling efficiency could also be implicated. %On.B is a measure of the amount of cortex that is remodeled; this would reflect a combination of osteoclast action at a given site (osteon size) and the number of sites that have undergone remodeling by BMUs (activation frequency). However, OPD more directly reflects activation frequency and, in this study, does not show significant evidence of heritability. This suggests that the parameter that is most susceptible to and reflective of genetic effects is osteon size, which biologically is dictated by osteoclast action at a given site. We interpret our present findings to indicate that normal, population-level variation in osteoclast-mediated cortical microstructure is significantly influenced by genes.

We are encouraged by the positive result of this first test for a genetic effect on intracortical remodeling-related microstructure, and we are now pursuing full-genome analyses of differentially expressed genes and differential gene exon usage between baboons that are discordant for bone strength for body size with the goal of identifying hubs within differentially active genetic networks that may provide novel targets for bone fragility treatment and prevention. We also plan to assess a larger suite of bone material and mechanical properties and biomechanical performance of the femur in a sample of baboons that is large enough to allow us to apply a powerful combined linkage and association approach that has been very successful in disease gene discovery for cardiovascular disease–related traits in these baboons.

This research program illustrates the value of nonhuman primate models, such as the baboon, for deciphering the dizzying array of genetic and environmental factors that culminate in a skeleton’s place along the bone strength/fragility continuum. Recent studies show that variation in bone structural integrity results from a complex relationship of coadaptation of traits that span all levels of bone’s hierarchical organization [42–45]. Inbred rodent studies have been instrumental in revealing that genes mediate the course of coadaptation of traits in response to the skeletal environment [42, 44]. However, the very inbreeding that makes these animals so valuable for localization of specific genetic effects and/or testing of functional hypotheses severely limits their utility in investigations of genetic regulation of population-level normal variation in outbred populations, such as humans. The absence of naturally occurring intracortical remodeling in rodents further limits the utility of rodents for studies of cortical bone remodeling variation that truly inform the human condition. Nonhuman primates can serve as an essential complementary model system in which to investigate the degree to which genetic variation underlies intracortical remodeling variation and the effect of such variation on skeletal fragility.
